# Roles of Prolyl Isomerases in RNA-Mediated Gene Expression

**DOI:** 10.3390/biom5020974

**Published:** 2015-05-18

**Authors:** Roopa Thapar

**Affiliations:** BioSciences at Rice-Biochemistry and Cell Biology, Rice University, Houston, TX 77251-1892, USA; E-Mail: rthapar@rice.edu; Tel.: +1-713-201-7875; Fax: +1-713-348-5154

**Keywords:** prolyl isomerase, cyclophilin, FKBP, parvulin, Pin1, histone mRNA, SLBP, KSRP, AUF1, HuR

## Abstract

The peptidyl-prolyl *cis-trans* isomerases (PPIases) that include immunophilins (cyclophilins and FKBPs) and parvulins (Pin1, Par14, Par17) participate in cell signaling, transcription, pre-mRNA processing and mRNA decay. The human genome encodes 19 cyclophilins, 18 FKBPs and three parvulins. Immunophilins are receptors for the immunosuppressive drugs cyclosporin A, FK506, and rapamycin that are used in organ transplantation. Pin1 has also been targeted in the treatment of Alzheimer’s disease, asthma, and a number of cancers. While these PPIases are characterized as molecular chaperones, they also act in a nonchaperone manner to promote protein-protein interactions using surfaces outside their active sites. The immunosuppressive drugs act by a gain-of-function mechanism by promoting protein-protein interactions *in vivo*. Several immunophilins have been identified as components of the spliceosome and are essential for alternative splicing. Pin1 plays roles in transcription and RNA processing by catalyzing conformational changes in the RNA Pol II C-terminal domain. Pin1 also binds several RNA binding proteins such as AUF1, KSRP, HuR, and SLBP that regulate mRNA decay by remodeling mRNP complexes. The functions of ribonucleoprotein associated PPIases are largely unknown. This review highlights PPIases that play roles in RNA-mediated gene expression, providing insight into their structures, functions and mechanisms of action in mRNP remodeling *in vivo*.

## 1. Roles of Peptidyl Prolyl Isomerases (PPIases) in mRNA Remodeling

Remodeling of messenger ribonucleoprotein (mRNP) complexes is a dynamic process that is essential for gene expression [1,2]. Although not well understood, the remodeling of mRNP assemblies by posttranslational modifications (PTMs) or proteins, such as helicases or peptidyl prolyl isomerases (PPIases), is necessary to change the composition of the mRNP as the mRNA is transcribed, processed, exported, translated, and subsequently degraded. In many ways, mRNP remodeling is analogous to chromatin remodeling [3,4]. Like histones and transcription factors that form and interact with DNA and chromatin, the interaction of RNA binding proteins with the RNA and protein components of mRNPs can be altered by PTMs or by recruitment of remodeling complexes. These remodeling complexes can act either by promoting the assembly or disassembly of the mRNP, or by targeting the mRNP to distinct cellular compartments or bodies. The PPIases form a unique family of proteins whose primary function has traditionally been thought of as a foldase or a chaperone. The scope of this review is to summarize the structural and functional characteristics of PPIases that are known to participate in remodeling RNA-protein complexes from the three major sub-families: the cyclophilins, FKBPs, and parvulins. The functional roles of most PPIases in RNA-mediated gene expression have not been well characterized.

## 2. Cyclophilin-Type PPIases that Participate in Pre-mRNA Splicing and Epigenetic Control of Transcription

The first cyclophilin to be discovered was CyPA [5], a small protein of 165 amino acids that is also the receptor for the immunosuppressant drug Cyclosporin A (CsA) [6]. The cyclophilins are well known for their enzymatic activity, *i.e.*, the ability to lower the rotational energy barrier about the prolyl imide bonds, thereby facilitating *cis-trans* proline isomerization and inducing conformational changes in their substrates [7,8]. However, contrary to this established view, not all cyclophilins are good PPIases, and some function only as chaperones by binding to their clients or substrates to stabilize a unique conformation, without catalyzing proline *cis-trans* isomerization. Furthermore, as has been shown for the cyclophilin PPIL1, cyclophilins can utilize surfaces outside the PPIase domains to promote protein-protein interactions in mRNP complexes. Several cyclophilins also have accessory domains, such as RRMs, U-box, TPR domains, and WD40 repeats [9] that are important for mediating protein-protein interactions.

**Figure 1:**
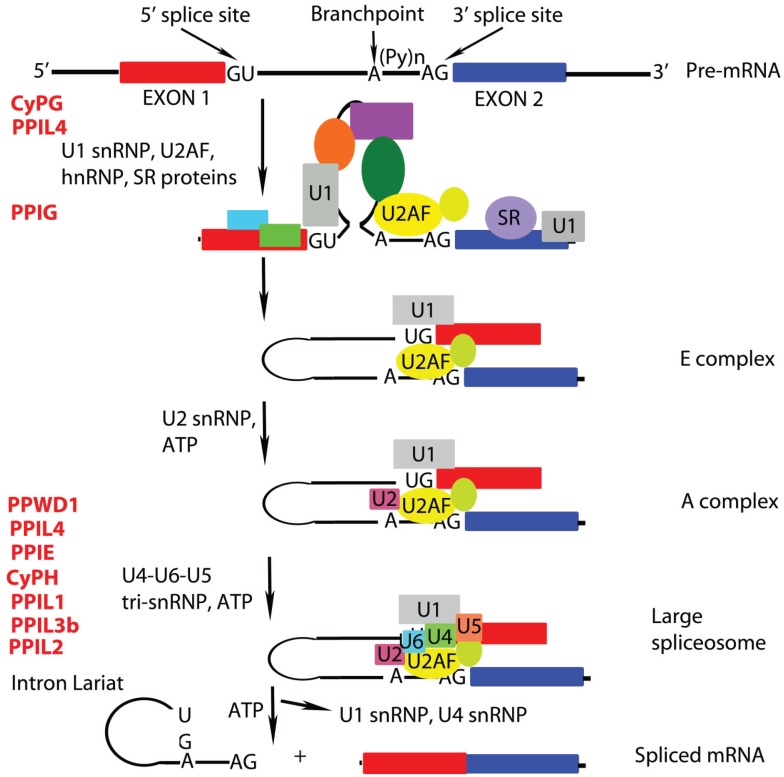
A simplified schematic of alternative splicing is shown. Splicing is directed by the GU dinucleotide at the 5' splice site of the intron and the AG nucleotide at the 3' splice site. The conserved branchpoint A nucleotide is located 20–50 nt upstream of the 3' splice site. The splicing reaction occurs in two transesterification steps and requires 5 snRNPs (U1, U2, U4, U5, and U6) that assemble on the pre-mRNA to form large macromolecular assemblies. The cyclophilins that are implicated in the different complexes are depicted.

X-ray crystal structures and solution NMR structures are available for cyclophilins from different species, in the unliganded form, as well as complexed to peptide ligands. Some of the structural features are highlighted in the sections below. Although most cyclophilins are non-essential proteins, they have received attention as drug targets in a spectrum of diseases due to their diverse roles in signaling and control of gene expression pathways. Eight cyclophilins that participate in RNA-mediated gene expression, and in particular pre-mRNA splicing (Figure 1) are highlighted in this section and are summarized in Table 1.

### 2.1 PPIL1 (also called CYPL1, hCyPX, CGI-124)

**Figure 2:**
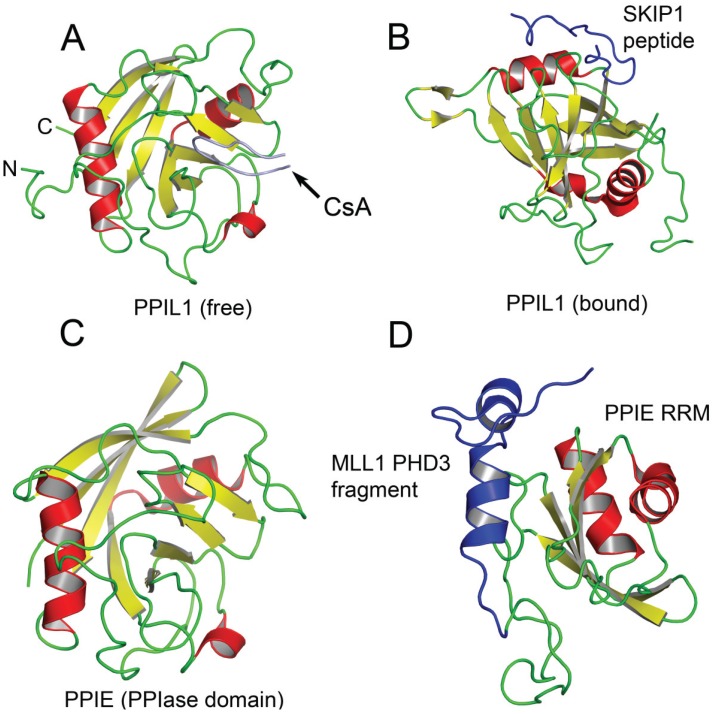
Structures of PPIL1 and PPIE free and complexed to spliceosomal proteins. In (**A**), the crystal structure of the free PPIase domain of PPIL1 is shown. The protein has a typical cyPA-like fold; In (**B**) the solution NMR structure of PPIL1 PPIase domain bound to the SKIP1 peptide is depicted. The SKIP1 peptide forms a hook like structure (in blue) and binds the PPIase domain at an allosteric site far removed from the active site; In (**C**), the crystal structure of the PPIase domain of PPIE is shown; In (**D**), the solution NMR structure of the MLL1-PHD3-PPIE-RRM complex is shown. The PHD3 fragment forms a helix that packs against the PPIE RRM.

The peptidyl prolyl isomerase-like protein 1 (PPIL1) [10] is a 166-residue cyclophilin with 41.6% sequence identity to cyclophilin A, that is an integral part of the 45 S activated B* spliceosome and the 35 S stable C complex of the spliceosome (Figure 1). PPIL1 is recruited by Ski-interacting protein (SKIP) [10], a protein that is involved in transcription and splicing, to form a high affinity complex that remains bound to the spliceosome C complex in 1M NaCl [11]. The PPIL1-SKIP complex plays an essential role in splicesosome activation as part of the Prp19 complex during the first catalytic step (B → B* transition) in the splicing reaction. Solution NMR [12,13] and X-ray crystallographic [14] studies reveal that PPIL1 has a typical cyclophilin fold consisting of an eight-stranded β-sheet, two α-helices and a short 3_10_ helix that pack against the β-sheet (PDB code 2X7K, Figure 2A). The root mean square deviation (r.m.s.d) over all backbone C^α^ atoms in secondary structure elements in the PPIL1 average NMR *vs.* CyPA crystal structures is 1.2 Å [12]. The active site geometry of PPIL1 is identical to cyclophilin A (CyPA) in the NMR and X-ray crystal structures. A notable difference between the PPIL1 and CyPA structures is that the C-terminal helix-α1 of PPIL1 is truncated by three residues, with the turn that links helix-α1 and the β3-strand adopting a different conformation than that observed in CyPA [12]. As a result, the loop that lies in proximity to helix-α1 (residues G65-Y78) also adopts a conformation that is different from that observed in CyPA. However, these structural differences around helix-α1 do not affect the PPIase activity of PPIL1. The protein exhibits PPIase activity with a *k_cat_/K_m_* of 4.2 × 10^6^ M^−1^·s^−1^, that is comparable to that of CyPA (*k_cat_/K_m_* of 14.6 × 10^6^ M^−1^·s^−1^) towards the substrate N-succinyl-Ala-Ala-Pro-Phe-p-nitroanilide. PPIL1 is also inhibited by cyclosporin A.

**Table 1:** Summary of cyclophilins involved in RNA-mediated gene expression.

Name in Review	Other Names	PDB Code/s	Proline Isomerase Activity?	Interacting Proteins in RNA Metabolism	Other Domains Present
PPIL1	CYPL1, hCyPX, CGI-124	1XWN (NMR) 2K7N (NMR) 2X7K (X-ray)	Yes	Ski-interacting protein (SKIP)	None
PPIL2	CYC4, Cyp60, UBOX7, Cyp58	1ZKC (ring-domain)	No	unknown	N-terminal U-box (E3 ligase)
PPIL3b	CyPJ	2OJU (X-ray) 2OK3 (X-ray) 1XYH (X-ray)	Unknown	Unknown protein in the U2snRNP	None
PPIE	CYP33, CYP-33	3UCH (X-ray PPIase) 2CQB (NMR-RRM) 2KYX (NMR-RRM) 3LPY (X-ray RRM) 3MDF (X-ray RRM) 2R99 (X-ray PPIase) 1ZMF (X-ray PPIase) 2KU7 (MLL1 PHD3-Cyp33 RRM chimeric protein)	Yes	MLL1 histone methyltransferase	N-terminal RRM
PPIL4	CyP57	None	Yes	RNA Pol II CTD	C-terminal RRM
PPWD1	CyP73	2A2N (X-ray PPIase)	Yes	Unknown	WD40 repeats
PPIH	Snu-Cyp20, USA-Cyp, CyPH	1MZW (X-ray PPIase)	Yes	hPrp4; hPrp18	None
PPIG	SR-Cyp, CARS-Cyp, CYPG, Matrin-CyP (rat)	2GW2 (X-ray PPIase)	Yes	Clk kinase; RNA Pol II CTD	N-terminal RS domains; Nopp140 repeats

The SKIP-PPIL1 interaction is of medium affinity *in vitro* and Surface Plasmon Resonance (SPR) experiments determined a binding constant (*K_D_*) of ~0.125 μM [12]. NMR chemical shift mapping and GST-pull down experiments initially showed that the binding interface involves residues distant from the PPIL1 active site (*i.e.*, strands β2, β7, and the loop preceding β5) and N-terminal residues 59–129 of SKIP [12]. SKIP1 and cyclosporin A can bind PPIL1 independently to distinct surfaces to form a stable ternary complex, indicating that the interaction of SKIP with PPIL1 does not require the PPIase activity of PPIL1. The solution NMR structure of the SKIP peptide-PPIL1 complex [13] shows that the SKIP peptide undergoes a disorder-to-order transition upon PPIL1 binding, wherein the bound SKIP peptide has a hook like extended structure (PDB code 2K7N, Figure 2B). The total buried surface area at the interface is 1622.4 Å^2^ and it involves 26 residues from PPIL1 and 19 residues from SKIP. The interface is predominantly hydrophobic, although electrostatic interactions also play a role in complex stability.

The precise biological role of human PPIL1 in facilitating the B → B* transition in the spliceosome is unclear. Since SKIP is an adaptor like protein that interacts with other spliceosomal proteins, the hypothesis is that PPIL1 may act as a chaperone or a PPIase to facilitate conformational changes in the 45 S activated spliceosome. Recently, PPIL1 from *Trypanosoma brucei* (*T. brucei*) has also been found to be part of a non-snRNP PRP19 complex that is comprised of PRP19, CDC5, PRL1, SPF27, PRP17, and SKIP as the core components [15]. This PRP19 complex is associated with the activated spliceosome in *T. brucei*. The identification of the PPIL1-SKIP complex in humans and trypanosomes indicates an essential role for this complex exists in spliceosome activation that is conserved in evolution.

### 2.2. PPIL2 (Also Called CYC4, Cyp60, UBOX7, Cyp58)

PPIL2 or Cyp60 is a large ~60 kDa (520 amino acids) protein that has a central cyclophilin-like domain and also has an N-terminal U-box domain that exhibits E3 ligase activity [16]. PPIL2 has polyubiquitination activity and shows greatest E3 ligase activity with Ubc2B or Ubc3 but is also active with UbcH7 [17]. PPIL2 may play functional roles in the nucleus and the cytoplasm. It interacts with CD147, an extracellular matrix metalloproteinase inducer [16], is involved in β-amyloid precursor (APP) processing and Alzheimer’s Disease [18], and was identified as part of the spliceosome by mass spectrometry [19]. Although it has been identified as a component of the spliceosome, the exact function of this cyclophilin in spliceosome assembly is unknown.

### 2.3. PPIL3b (Also Called CyPJ)

The PPIL3 gene produces two alternatively spliced cyclophilin like proteins PPIL3a and PPIL3b, both of which are ubiquitously expressed in human tissues [20]. PPIL3b has been identified as part of the B2 complex of the spliceosomes (but not B1) [21]. It is likely to be bound to the U2 snRNP, although the roles of both PPIL3a and PPIL3b are unknown. Human PPIL3b (or CyPJ) is about 50% identical to hCyPA and consists of 160 amino acids. The 2.6 Å crystal structure of CyPJ has been solved and shows a cyclophilin like fold with eight β-strands and four α-helices [22]. The r.m.s.d between the backbone C^α^ atoms of CyPJ and CyPA is 1.8 Å. There are small differences between the two proteins that correspond to surface insertions/deletions in four turns and loops. The most notable difference is a longer loop that is present corresponding to the segment Pro135-Asn145 that is stabilized by three backbone-backbone H-bonds. In addition CyPJ has a unique disulfide bridge between Cys18 and Cys25 that is not present in CyPA The active site of CyPJ is identical to that of CyPA.

### 2.4. PPIE (Also Called CYP33, CYP-33)

PPIE or Cyp33 is a nuclear cyclophilin that has an N-terminal RNA recognition motif (RRM) that can bind poly (A) and poly (U) tracts of RNA, but not poly (G) and poly (C) RNAs [23]. RNA binding stimulates the PPIase activity of PPIE *in vitro* [23]. PPIE was first isolated from human T cells as a protein of 301 amino acids [24]. The protein had PPIase activity and was inhibited by CsA [24]. The 1.88 Å crystal structure of the PPIase domain of PPIE confirms a typical cyclophilin fold consisting of an eight stranded β-barrel with two α-helices that pack against the β-sheet (PDB code 1ZMF, Figure 2C) [25]. The overall r.m.s.d between the backbones of PPIE and CyPA PPIase domains is 0.58 Å. The protein has been identified as a component of the spliceosome by proteomic approaches [19], however its function in splicing is undetermined. Intriguingly, PPIE is involved in the epigenetic regulation of transcription by directly association with the PHD3 finger of the Mixed Lineage Leukemia 1 (MLL1) histone methyltransferase [26,27]. MLL1 is a transcriptional reader and writer and binds to the N-terminus of histone 3 (H3) trimethylated at lysine 4 (H3K4me3). Over-expression of PPIE represses transcription of MLL1 target genes, such as *HOXC8*, *HOXA9*, *CDKN1B*, and *C-MYC*, by directly altering H3K4 methylation and H3 acetylation at the promoter regions of these genes [28]. PPIE proline isomerization activity is important for transcription repression [28]. Therefore, PPIE plays an active role in epigenetic control of transcription. PPIE introduces a conformational change in MLL1 by catalyzing proline *cis-trans* isomerization about the His1628-Pro1629 peptide bond that lies in the linker sequence between the PHD3 finger and the bromeodomain of MLL1 [28]. Proline isomerization allows the PPIE RRM to bind the PHD3 finger of MLL1 (PDB code 2KU7, Figure 2D) [29], promoting the recruitment of histone deacetylases to MLL1, which in turn represses transcription [26]. Solution NMR and crystal structures of the PPIE RRM domain show a typical α/β RRM fold [29]. NMR studies reveal that although the binding sites of the PPIE RRM and H3K4me3 on the PHD3 finger do not overlap, binding of either partner (PPIE RRM or H3K4me3) inhibits binding of the other [28]. The PPIE RRM can displace MML1 from H3K4me3 [28]. The role of RNA in this unique mechanism of epigenetic regulation via PPIE remains unknown, although NMR chemical shift mapping and mutagenesis experiments show that the binding sites on PPIE for RNA and the PHD3 finger overlap [28,29].

### 2.5. PPIL4 or CyP57

PPIL4 is another RRM containing cyclophilin that has been found to be a component of the spliceosome [19,30]. It consists of 492 amino acids and the RRM domain is found C-terminal to the PPIase domain. However, in contrast to PPIE, RNA binding inhibits the PPIase activity of the PPIL4 ortholog from *A. thaliana* (*At*Cyp59) [31]. *At*Cyp59 also binds the RNA Pol II Carboxy-terminal domain (CTD), facilitating in its dephosphorylation and slowing cell growth [32].

### 2.6. PPWD1 (Also Called CyP73)

PPWD1 is a large PPIase that has a C-terminal CyPA-like domain and has four WD40 repeats in the N-terminal domain. It has been found to be a component of the human spliceosome C complex [19,30]. The crystal structure of the PPIase domain of human PPWD1 has been determined at 1.65 Å resolution [33]. PPWD1 shares ~60% sequence similarity with CyPA. Consistent with this, the backbone C^α^ atoms of PPWD1 and CyPA have an r.m.s.d of 1.34 Å over 124 atoms. The main structural difference between the isomerase domains lies in the β1-β2 loop, which is shorter by five residues in PPWD1 as compared to CyPA. Intriguingly, the crystal structure reveals intermolecular interactions of the PPIase domain with an internal Gly-Pro peptide from another PPWD1 molecule in the asymmetric unit that binds in a *trans*-Pro conformer. PPWD1 is able to bind this peptide, but kinetic measurements demonstrate that it does not exhibit PPIase activity towards this peptide [33]. In contrast, PPWD1 does exhibit PPIase activity against other standard substrates, such as succinyl-Ala-Ala-Pro-Phe-p-nitroaniline [33]. The sequence and structural determinants of substrate binding *vs.* catalysis of PPWD1 are not yet understood. The role/s of the accessory WD40 domain in mediating protein-protein interactions in the spliceosome are unclear.

### 2.7. PPIH (Also Called Snu-Cyp20, USA-Cyp, CyPH)

Cyclophilin H (CyPH) or PPIH was first reported to be a component of the U4/U6 small nuclear ribonucleoprotein particle (snRNP) by Lührmann and colleagues in 1998 [34]. PPIH is a 19 kDa protein with a single cyclophilin domain that has PPIase activity and mutagenesis studies [35,36] along with the crystal structures [37,38] are consistent with an active site that is similar to other members of the cyclophilin family. However, the PPIase activity of PPIH is only ~15% compared to that of CyPA [35]. A *k_cat_/K_M_* value of 8.8 × 10^6^ M^−1^·s^−1^ was measured for CyPA and a *k_cat_/K_M_* of 1.3 × 10^6^ M^−1^·s^−1^ for PPIH towards the same tetrapeptide substrate [35]. PPIase activity over all cyclophilins can vary over two orders of magnitude and is dependent on both the affinity towards the substrate, as well as the intrinsic catalytic activity of the cyclophilin. Mutation of active site residues that are important for catalysis completely abrogates PPIase activity of PPIH, consistent with it being a prolyl isomerase.

The 2.0 Å crystal structure of PPIH [38] shows that PPIH has a CyPA-like α/β fold with an eight-stranded β-sheet and two α-helices that pack against the β-sheet. In addition PPIH has two 3_10_-helices, one that lies in the loop connecting strands β6 and β7 that is present in CyPA, and the second in a loop between helix α2 and strand β8 that is absent in CyPA. In addition, PPIH has a longer *N*-terminus (by seven amino acids), an extra amino acid in the loop connecting helix α2 and strand β8, and five additional amino acids in the loop connecting helix α1 and strand β3, compared to CyPA. The presence of an additional amino acid in the loop between helix α2 and strand β8 results in an altered conformation for residues in the loop, which adopts a short 3_10_-helix. The presence of five amino acids in the loop connecting helix α1 and strand β3 introduces a binding cleft in the protein that is predominantly hydrophobic and may be important for protein-protein interactions. The active site superimposes perfectly with that of CyPA and the root mean square deviation (r.m.s.d) for 158 shared backbone C^α^ atoms of PPIH and hCyPA is 0.8 Å.

PPIH interacts specifically with hPrp3 and hPrp4 (or U4/U6-60K) [34] and the non-snRNP protein hPrp18 [36] to form two distinct complexes (Prp3/Prp4/PPIH and PPIH/Prp18) during splicing. Whereas Prp3 and Prp4 are incorporated into the U4/U6 *di*-snRNP, Prp18 is involved in the assembly of the tri-snRNP (U4/U6.U5) and plays a role in the second trans-esterification reaction. hPrp4 and hPrp18 have a 31 amino acid region that is highly homologous in sequence and this sequence and binds PPIH in both proteins [36]. In hPrp4, this region comprises residues 107–137 and lies N-terminal to the WD-repeat elements. In hPrp18, this region comprises residues 83–114. A 2.1 Å crystal structure of PPIH complexed to a 31 residue peptide corresponding to the hPrp4 binding site has been determined [37]. No large-scale conformational changes are observed in PPIH upon binding the hPrp4 peptide and the r.m.s.d over 171 backbone C^α^ atoms of free and bound PPIH is 0.56 Å. The peptide binds a region on PPIH that is remote from the PPIH active site. The equilibrium dissociation constant (*K_D_*) for the PPIH-hPrp4 peptide was determined to be 1.97 μM by isothermal titration calorimetry. The hPrp4 peptide is disordered in the absence of PPIH but folds into short α-helices (α1 and α2) that are connected by a loop that has a short β-strand (β1) in the crystal structure of the complex. The β-strand of hPrp4 (residues Ile118-Phe121) interacts with strand β8 of PPIH via backbone H-bonds, thereby extending the β-sheet of the cyclophilin. The total buried surface area between PPIH and hPrp4 peptide is 878 Å^2^. The side chain of Phe121 of the hPrp4 peptide that lies at the edge of the β-strand inserts into a hydrophobic cavity of PPIH formed by Pro57, Ile58, Gly59, Tyr60, and Lys61. The structure reveals that PPIH interacts with other spliceosomal proteins, such as hPrp4 and hPrp18 utilizing a surface that is removed from the active site, thereby leaving the enzyme catalytically active to facilitate prolyl *cis-trans* isomerization on its substrates.

### 2.8. PPIG (Also Called SR-Cyp, CARS-Cyp, CYPG, Matrin-CyP (Rat))

PPIG or Clk associating RS-cyclophilin (CARS-Cyp) was identified in a yeast two-hybrid screen using the Clk (CDC28/cdc2-like kinase) as a bait in 1996 [39]. Clk kinase plays an important role in pre-mRNA splicing by phosphorylating arginine/serine rich (RS) splicing factors. PPIG has a CyPA-like domain at the N-terminus, RS domains in the C-terminus, and two Nopp140 repeats that are important for nuclear import in the middle of the protein. It is a large, 89 kDa (754 amino acids) cyclophilin [39]. PPIG is expressed ubiquitously in several tissues [39]. The rat isoform of PPIG is Matrin-CyP which is ~93% homologous to human PPIG and was identified in 1998 [40] as a protein that was enriched in the nuclear matrix and co-localized with RNA splicing factors in nuclear speckles during Interphase. It is also a large 88 kDa protein with an N-terminal cyclophilin domain that is followed by an acidic region, and three serine/arginine-rich (SR) regions that are commonly found in splicing factors and are hyperphosphorylated [40]. Confocal microsopy experiments showed that Matrin-CyP colocalized with the 70 kDa subunit of the U1 snRNP [40]. During mitosis, Matrin-CyP was found to redistribute in the nucleus and associated with non-snRNP SR proteins [40]. Matrin-CyP is a functional PPIase with a 16-fold lower *k_cat_/K_M_* compared to CyPA [40]. Its PPIase activity is also inhibited by CsA. The precise role/s of Matrin-CyP in regulating splicing has not been elucidated.

PPIG has been shown to interact directly with the phosphorylated RNA Pol II Carboxy-terminal domain (CTD) via its RS domains *in vivo* and *in vitro* [41]. Since the phosphorylated CTD is known to be associated with nuclear speckles and is also important for splicing, PPIG may play an active role in transcription elongation, as well as splicing.

## 3. FK506 Binding Proteins (FKBP) Involved in Epigenetic Silencing and mRNA Stability

The immunosuppressive drugs FK506 and rapamycin that are used in organ transplantation bind protein receptors called FK506 binding proteins (FKBPs) in T-cells [42,43,44,45]. FKBPs are peptidyl prolyl *cis-trans* isomerases that participate in protein trafficking, signaling, and transcription as foldases or chaperones as their normal function in the cell. However these receptors act by a gain-of-function mechanism when bound to drugs, binding the primary member of the FKBP family, FKBP12. The FKBP12-FK506 complex inhibits the activity of a calmodulin-dependent protein phosphatase, calcineurin [46], whereas the FKBP12-rapamycin complex targets the phosphatidylinositol kinase-related kinases FRAP, RAFT1, and mTOR complexes [47] that are required for translation and cell cycle regulation. Of the 18 FKBPs in the human genome, at least three are known to participate in posttranscriptional control of gene expression.

**Figure 3:**
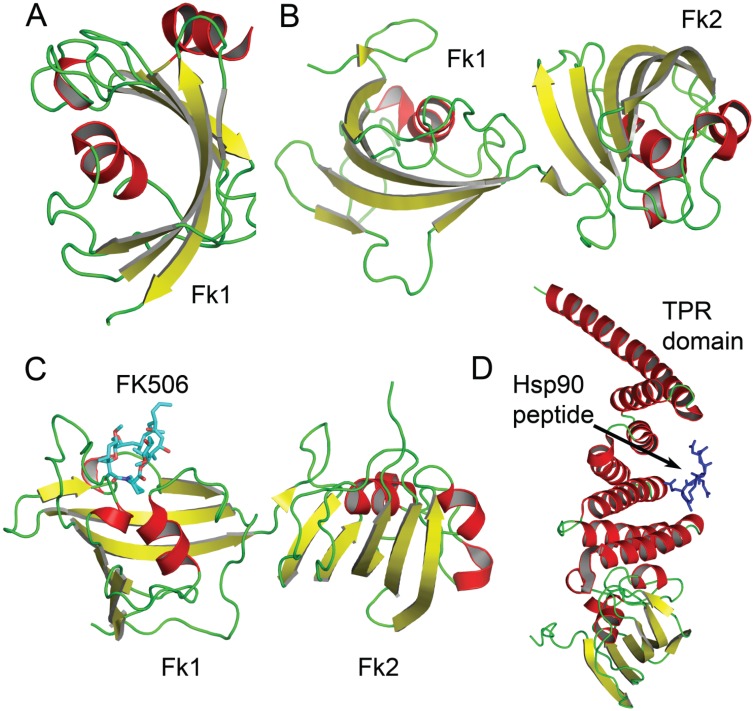
Structures of FKBP domains free and complexed to FK506 and a Hsp90 peptide. In (**A**), the crystal structure of the free Fk1 (active PPIase domain) of FKBP51 is shown. The Fk1 domain fold is conserved in all FKBPs; In (**B**) the crystal structure of the tandem Fk1-Fk2 domains of FKBP52 is depicted; In (**C**), the crystal structure of the tandem Fk1-Fk2 domains of FKBP52 bound to FK506 is shown; In (**D**), the interaction of the C-terminal TPR domain of FKBP52 with a Hsp90 pentapeptide (shown in blue) is depicted.

Human FKBP51 and FKBP52 show 55% sequence identity and have a common domain structure consisting of an N-terminal FKBP domain (Fk1), and FKBP-like domain (Fk2), and a TPR domain at the C-terminus. Several X-ray crystal structures and solution NMR structures of FKBP domains are now available (Table 2). All FKBP domains consist of a curved five-stranded antiparallel β-sheet that wraps around a short α-helix (Figure 3A). This core structure binds the substrate proline and is preserved in all structures. An additional α-helix is present in the Fk1 domain of FKBP51 (PDB code 3O5I) [48], but this region is disordered in the crystal structure of FKBP52 (PDB code 1Q1C, Figure 3B) [49]. The PPIase activity is limited to the Fk1 domain and no PPIase activity is observed for the Fk2 domain of FKBP52 [49]. Structural differences between the different FKBP proteins are localized to peripheral regions outside the core FK1 domain. The inhibitor FK506 is bound in a hydrophobic pocket near the central α-helix. The FK506 ligand is involved in an extensive network of hydrogen bonding interactions with the Fk1 domain as well as aromatic stacking interactions with Trp, Tyr, and Phe residues (Figure 3C). The crystal structure of the C-terminal TPR domain of FKBP52 (PDB code 1QZ2) bound to an Hsp90 peptide illustrates how accessory domains participate in mediating protein-protein interactions (Figure 3D).

### 3.1. FKBP4 and FKBP5

FKBP 4 and 5 have been identified as novel components of the RNA-induced silencing (RISC) loading complex in embryonic stem cells [50] and HeLa cells [51]. These FKBPs directly associate with Ago2, and inhibition of this interaction by FK506 destabilizes Ago2, leading to decreased cellular protein levels of Ago2. Since FKBP 4/5 also associate with Hsp90, an established component of RISC, the model is that these FKBPs act as co-chaperones, stabilizing Ago2, as well as assisting Hsp90 mediated loading of Ago2 with siRNAs and miRNAs. Knockdown of FKBP4 decreases the efficiency of RNAi. FK506 treated cells also show decreased miRNA expression, consistent with the proposed role of FKBP 4 and 5 as important chaperones that stabilize the RISC complex.

**Table 2:** Summary of FKBPs involved in RNA-mediated gene expression.

Name in Review	Other Names	PDB Code/s (for PPIase Domain Containing Structures)	Proline Isomerase Activity?	Interacting Proteins in RNA Metabolism	Other Domains Present
FKBP4	FKBP51 FKBP52 FKBP59	1Q1C (X-ray); 4DRJ (X-ray); 1QZ2 (X-ray); 4TW8 (X-ray); 1ROU (NMR); 1ROT (NMR); 1N1A (X-ray); 4LAY (X-ray); 4LAX (X-ray); 4LAW (X-ray); 4LAV (X-ray); 1P5Q (X-ray)	Yes	Hsp90, Ago2	C-terminal TPR
FKBP5	FKBP51 FKBP54	3G6P (X-ray); 3G6Q (X-ray); 3G6R (X-ray); 3G6T (X-ray); 3G6U (X-ray); 1KT0 (X-ray); 1KT1 (X-ray); 4TW6 (X-ray); 4TXO (X-ray); 3O5D (X-ray); 3O5E (X-ray); 3O5G (X-ray); 3O5I (X-ray); 3O5J (X-ray); 3O5K (X-ray); 3O5L (X-ray); 3O5M (X-ray); 3O5O (X-ray); 3O5P (X-ray); 3O5Q (X-ray); 3O5R (X-ray); 4DRK (X-ray); 4DRM (X-ray); 4DRN (X-ray); 4DRO (X-ray); 4DRP (X-ray); 4DRQ (X-ray); 4JFI (X-ray); 4JFJ (X-ray); 4JFK (X-ray); 4JFL (X-ray); 4JFM (X-ray); 4TW7 (X-ray); 4W9O (X-ray); 4W9P (X-ray); 4W9Q (X-ray); 4DRH (X-ray); 4DRI (X-ray);	Yes	Hsp90, Ago2, Akt	C-terminal TPR
FKBP6	FKBP36	3B7X	No	Hsp90, Hsp27	C-terminal TPR
FKBP25	FKBP3	3KZ7 (X-ray); 1PBK (X-ray); 4JYS (X-ray); 2KFV (NMR)	Yes	HDAC1, HDAC2, YY1, CK2, Nucleolin, HMG II	Extended N-terminus

### 3.2. FKBP6

Similar to FKBP4/5 above, FKBP6 associates with Hsp90 via its tetratricopeptide repeat (TPR) domain to deliver piRNAs to the Piwi protein Miwi2 in mice [52]. Piwi proteins are Argonautes that are expressed only in gonads and they associate with Piwi-interacting RNAs (piRNAs), which are 24–31 nucleotides (nt) long. The Piwi proteins and piRNAs are involved in epigenetic silencing of transposable elements. Although they are small RNAs, piRNAs have a unique mechanism of RNA processing that is different from the Dicer-dependent processing of miRNAs. The mature piRNAs import Miwi2, which is required for methylation of transposon promoters on DNA. FKBP6 has been shown to be essential for transposon silencing and DNA methylation. Mice lacking *Fkbp6* show male infertility. Surprisingly, the N-terminal PPIase domain of FKBP6 lacks prolyl isomerase activity, and does not interact with FK506, even though the overall fold of the PPIase domain is similar to that of the “active” FKBP12. The crystal structure shows that this active site of FKBP6 is quite different from FKBP12, explaining the lack of observed isomerization activity in enzyme assays. FKBP6 interacts with Hsp90 in mouse testes extracts, as well as HEK293T cells, and this interaction is dependent on the C-terminal TPR domain. The biological data implicate a role for FKBP6 in piRNA biogenesis via interaction with Hsp90, although the exact mechanism is not clear [52].

### 3.3. FKBP25

FKBP25 is a nuclear PPIase that participates in epigenetic regulation of gene expression, as well as ribonucleoprotein complexes. It is known to directly associate with the histone deacetylases HDAC1 and HDAC2 [53] via the extended N-terminus. It also associates with the transcription factor Yin-Yang (YY)1 [53]. The yeast orthologue of FKBP25, called Fpr4, binds the nucleosome and exhibits both histone chaperone and histone PPIase activities [54,55]. Recent evidence from fractionation experiments [56] and proteomics studies [57] suggests that FKBP25 is present in both the nucleus and the cytoplasm and is a component of spliceosomal complexes as well as polyribosomes [56]. FKBP25 was found to interact with the immature large ribosomal subunit in nuclear extracts and likely plays a role in ribosome biogenesis by acting as a protein chaperone in preribosomes, although its precise function is unknown [57].

## 4. Parvulins Involved in Regulating Transcription and mRNA Turnover

Parvulins form the third family of PPIases that are conserved in prokaryotes and eukaryotes (Table 3). Parvulins are small PPIases (~10–20 kDa) that have a preference for hydrophobic residues preceeding the proline. *E. coli* Par10, the founding member of this sub-family is ~10 kDa consisting of only the PPIase domain. In humans, the Parvulin family of *cis-trans* prolyl isomerases includes Pin1, Par14, and Par17. Pin1 is the most well characterized parvulin and is a drug target for treatment of cancer, Alzheimers disease, asthma, and inflammation. Pin1 regulates the protein and mRNA levels of several proto-oncogenes, cytokines, chemokines, and tumor suppressors implicated in disease. Pin1 can alter gene expression by affecting transcription or mRNA stability. It has also been implicated in regulating protein levels by altering the sub-cellular localization and ubiquitination of protein targets. The role of Pin1 in posttranscriptional mechanisms that control gene expression is summarized in greater detail below. In addition to Pin1, there is an additional locus in the human genome on Chromosome Xq13 that encodes two other parvulins: Par 14 and Par 17. Par14 and Par17 are less well characterized biologically, but have been implicated in chromatin remodeling and cell proliferation.

### 4.1. Pin1

The peptidyl prolyl isomerase Pin1 is a regulator of the cell cycle and is required for progression of cells into mitosis [58,59,60]. Pin1 has been shown to catalyze *cis-trans* isomerization of proline residues in proteins that have a phosphorylated serine or threonine preceeding a proline. Pin1 frequently acts along with kinases and phosphatases to induce conformational changes in its target proteins thereby regulating their activity. Pin1 protein targets include several oncogenes and transcription regulators such as c-Myc [61], c-Jun [62], p53 [63,64], beta-catenin [65], as well as numerous cell cycle regulators that include Raf-1 kinase [66] and Cyclin-E [67] and several RNA-binding proteins that play important roles in regulation of signaling and gene expression (discussed below). Pin1 could be a useful a biomarker in human cancers and is a target for drug therapy [68,69,70]. Pin1 regulates transcription and pre-mRNA processing [71,72,73] by interacting with the C-terminal domain (CTD) of RNA Polymerase II. Recently, the yeast orthologue of Pin1 (Ess1) has also been implicated to play a role in transcription termination of snRNAs and mRNAs with short ORFs [74,75,76].

**Table 3:** Summary of parvulins involved in RNA-mediated gene expression.

Name in Review	Other Names	PDB Code/s (for PPIase Domain Containing Structures)	Proline Isomerase Activity?	Interacting Proteins in RNA Metabolism	Other Domains Present
Pin1	DOD, UBL5 Ess1 (yeast)	1PIN (X-ray), 1NMV (NMR) 1NMW (NMR), 1F8A (X-ray) 2ITK (X-ray), 4TYO (X-ray) 2F21 (X-ray), 3TDB (X-ray) 3TCZ (X-ray), 3WH0 (X-ray) 3KAG (X-ray), 3KAI (X-ray) 3KCE (X-ray), 3KAH (X-ray) 3KAC (X-ray), 3KAB (X-ray) 3KAD (X-ray), 3KAF (X-ray) 1ZCN (X-ray), 2RUC (NMR) 2RUD (NMR), 2Q5A (X-ray) 3I6C (X-ray), 3ITK (X-ray) 3JYJ (X-ray), 3ODK (X-ray) 3IK8 (X-ray), 3IKD (X-ray) 3IKG (X-ray), 2ZQS (X-ray) 2ZQT (X-ray), 2ZQU (X-ray) 4U96 (X-ray), 4QIB (X-ray) 4TNS (X-ray), 4U84 (X-ray) 4U85 (X-ray), 2ZR4 (X-ray) 2ZR5 (X-ray), 2ZR6 (X-ray) 1YW5 (Ess1)	Yes	AUF1 KSRP HuR SLBP RNA Pol II CTD	WW
Par14	PIN4	1EQ3 (NMR) 1FJD (NMR) 3UI4 (X-ray 0.8 Å) 3UI5 (X-ray 1.4 Å) 3UI6 (X-ray 0.89 Å w/oxidized DTT)	Yes	Unknown	N-terminal basic domain
Par17	PIN4	Same as above. Par17 is related to Par14	Yes	Unknown	N-terminal basic domain + helical extension

The functional role of Pin1 in modulating transcription via interaction with the RNA Pol II CTD has been particularly well-studied. The first insight into the role of Pin1 in interaction with the RNA Pol II CTD came from the yeast orthologue of Pin1 called Ess1 [77,78]. Temperature sensitive mutations in Ess1 resulted in transcription readthrough of a number of genes due to impaired 3' end processing. Additional studies showed that Ess1 directly associated with the phosphorylated CTD [79] and genes such as *RPB7* and the CTD phosphatase *FCP1* could rescue the growth defects of the Ess1 temperature sensitive mutations [79], when present at high copy-number. Proteomics studies have shown that Ess1 binds a phosphorylated CTD affinity column, providing additional evidence for direct association [80]. These studies provided the first evidence that Ess1 could modulate RNA Pol II CTD function via direct interaction and possibly isomerization of the RNA Pol II CTD.

In mammals, Pin1 has also been shown to interact with RNA Pol IIO [81] and can modulate RNA Pol II CTD phosphorylation [82]. The CTD consists of 52 heptad repeats of the sequence YSPTSPS [82,83,84]. The CTD is phosphorylated at Ser2 by CDK12 [85] and/or CDK9 [86,87] and dephosphorylated by Ssu72 and/or Fcp1 [88,89]. Phosphorylation at this site recruits the histone methyltransferase complex Set2 and activated transcription. Phosphorylation at Ser5 by CDK7 facilitates the dissociation of RNA Pol II from the pre-initiation complex and elicits promoter escape [90]. Ser5 phosphorylation also promotes recruitment of splicing and capping factors [91], as well as the Set1 methyltransferase complex MLL and histone deacetylase complexes, such as Set3C [92,93,94,95]. Mutations in Ess1 have been shown to increase Ser5 phosphorylation levels [74,75,96]. Ess1 has been shown to preferentially bind the p-Set5 over p-Ser2 form of the CTD and stimulate dephosphorylation of p-Ser5 by Ssu72 *in vitro* and *in vivo* [97]. This dephosphorylation requires the isomerization activity of Ess1. Phosphorylation at Ser7 occurs early in transcription by CDK7. Ser7 is also dephosphorylated by Ssu72 [96]. Ess1 is required for Ser5 and Ser7 dephosphorylation by Ssu72 [96]. It does not appear to be essential for Ser2 dephosphorylation. Therefore, Ess1 stimulates dephosphorylation of the RNA Pol II CTD, playing a critical role in regulating the CTD during the transcription cycle. These studies on both Ess1 and Pin1 indicate that Pin1 acts in transcription initiation and pre-mRNA processing by controlling the association of RNA Pol II with active genes and by modulating CTD phosphorylation. Pin1 functions early in the transcription cycle, during initiation, and not during elongation [81]. The crosstalk between proline isomerization and phosphorylation of the RNA Pol II CTD exemplifies how the activity of kinases and phosphatases can be controlled by prolyl isomerases that act to alter the isomerization state of the adjacent proline. This is illustrated by a recent study in which the effect of Pin1 on dephosphorylation by the phosphatases Scp1 and Ssu72 was examined [98]. Pin1 rapidly converted trans-Pro to *cis*-Pro in this study, leading to an apparent increase in the activity of the Ssu72 phosphatase, which is specific for *cis*-Pro containing phosphopeptides. In contrast, the phosphatase activity of Scp1 was not significantly affected by Pin1, since Scp1 recognizes only the *trans*-Pro isomer. Therefore, Pin1 can affect the response of phosphatases *in vivo* by regulating the conformation of the CTD by proline isomerization.

**Figure 4:**
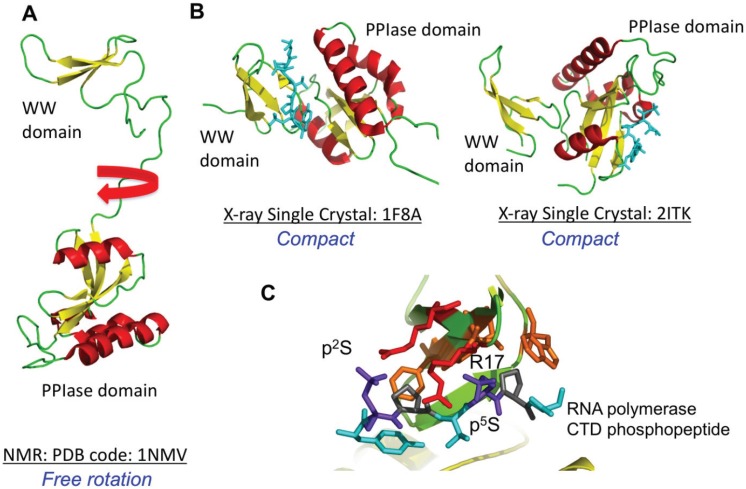
NMR and X-ray crystal structures of Pin1 free and bound to peptides are shown. In (**A**), the solution NMR structure (PDB code 1NMV) is depicted showing the Pin1 PPIase domain and the WW domain separated by a linker; In (**B**) two crystal structures of Pin1 bound to phosphopeptides are shown. In the first structure (PDB code 1F8A), the peptide interacts with the WW domain and in the second complex (PDB code 2ITK), the peptide interacts with the PPIase domain; In (**C**), the interactions of the WW domain with a doubly phosphorylated Ser-Pro peptide is shown. The phosphoserines are shown in blue and the arginine side chains from the WW domain are shown in red.

Numerous X-ray and NMR structures of full-length Pin1 and its sub-domains have been deposited in the Protein Data Bank (Table 3), either free or bound to peptide ligands and inhibitors. Pin1 has a two-domain structure consisting of an N-terminal WW domain and a C-terminal PPIase domain (Figure 4) [99,100]. Solution NMR studies show that the WW and PPIase domains rotate independently with different correlation times (Figure 4A) [101,102]. This is in contrast to crystal structures of Pin1 solved in the presence of peptide ligands where the phosphopeptide binds either the WW domain or the PPIase domain, but the two domains pack against each other to adopt a compact structure (Figure 4B) [100]. The mechanism by which Pin1 interacts with phosphoproteins to catalyze *cis-trans* prolyl isomerization therefore is not clear. Pin1 is capable of a bivalent interaction with its substrates [103] in which both the WW domain and the PPIase domain can interact independently with the substrate, suggesting it may be capable of interacting with two phosphoproteins or two phosphorylated S/T-P sites in the protein at the same time. The preferred substrate sequence for Pin1 is X-P-X-pS/T-P.

Several recent studies indicate that the prolyl isomerase Pin1 regulates mRNA stability for a subset of eukaryotic mRNAs. Pin1 has been implicated in control of mRNA stability and turnover of the cytokine GM-CSF [104,105,106], P*th* [107,108], and TGFβ [109] mRNAs, all of which have AU-rich *cis*-elements (ARE) in their 3' untranslated regions (3' UTRs) and play important roles in control of the immune response in inflammation and asthma. These ARE sequences bind ARE binding proteins (ARE-BPs) such as AUF1 and KSRP, that regulate mRNA turnover. Pin1 regulates mRNA turnover of GM-CSF mRNAs by binding AUF1 that is phosphorylated by ERK at Ser83-Pro84 so as to dissociate AUF1 from the GM-CSF mRNA, preventing AUF1-mediated mRNA degradation [106]. The Ser181-Pro182 sequence in the ARE-BP KSRP is also targeted by Pin1, for isomerization and dephosphorylation [108,110]. Pin1-mediated KSRP dephosphorylation triggers decay of P*th* mRNA *in vivo* [107,111]. Other ARE-BPs such as HuR may also be Pin1 targets *in vivo* [112]. We recently showed that Pin1 also regulates histone mRNA decay by controlling the dissociation of Stem-Loop Binding Protein (SLBP) from the histone mRNA [113], a rate limiting step in regulation of histone mRNA turnover. Pin1 binds the HPR(phospho)TPNK sequence that is conserved in the l-motif RNA-binding domain of all SLBPs [113,114] and undergoes prolyl isomerization [113,115], although efficient binding to Pin1 also requires a phosphodegron sequence (Ser20, Ser23) in the *N*-terminus of SLBP [113]. The Pin1-SLBP interaction also regulates nucleocytoplasmic shuttling of phosphorylated SLBP as well as SLBP ubiquitination [113].

In addition to these targeted studies, Pin1 gene silencing combined with microarray based gene expression profiling and quantitative RT-PCR shows that cellular pathways, namely cell adhesion, phosphatidylinositol signaling, and leukocyte migration are predominantly perturbed in response to Pin1 gene silencing [112]. All core histone mRNAs are stabilized in response to a Pin1 knockdown [112]. In addition, this study identified 78 oncogenes and cell cycle regulators that have AU-rich elements (AREs) in their 3' UTRs and whose abundance was linked to Pin1 expression. Intriguingly, several genes that showed altered abundance in the presence of a Pin1 knockdown had short half-lives (<4 h). Therefore, the data implicate Pin1 as being an important regulator of mRNA turnover by modulating the activity of RNA binding proteins.

### 4.2. Par14 and Par17

Par14 has a C-terminal parvulin-like PPIase domain and an N-terminal basic domain whereas Par17 has a longer N-terminal helical extension compared to Par14. The PPIase domains of Par14 and Par17 have a five amino acid insertion near the C-terminus (between the third β-strand and the C-terminal α-helix) that is not present in Pin1. Par14 can partially compensate for Pin1 depletion in mouse endothelial fibroblasts (MEFs) [116] in cell cycle regulation [116] and chromatin remodeling [117]. In addition, unlike Pin1, Par14 is not specific for phosphoproteins and it lacks the phosphate-binding loop present in Pin1. The solution NMR structure of Par14 (PDB codes 1EQ3 and 1FJD) [118,119] reveals that the Par14 PPIase domain is closer in structure to *E. coli* Par10 than Pin1. The five amino acid insertion forms a loop that lies ~10 Å away from the active site and, therefore, is not likely to be involved in substrate recognition.

Active site residues that are important for catalysis in Pin1 are different in Par14. In particular, the nucleophile C113 in Pin1 that plays an important role in catalysis is an aspartic acid (Asp74) in Par14. A recent 0.8 Å crystal structure of Par14 [120] implicates a catalytic tetrad comprised of Asp74, His42, His123, and Thr118 in the Par14 active site as being important for catalytic activity of all parvulins. A hydrogen-bonding network is clearly visible at subatomic resolution between Asp74-O—H-N_ε2_-His42-N_δ1_—H-N_δ1_-His123-N_ε2_—HO-Thr118. The hydrogen-bonding pattern is consistent with hydrogen bonds reported for the two histidines in a recent NMR study of the related parvulin PrsA from *S. aureus* [121]. The rotamers and tautomeric states observed in the crystal structure are confirmed by pKa and activity measurements, and suggest that a Cys/Asp-His-His-Thr/Ser tetrad is essential for catalytic activity of all parvulins.

Although Par14 and Par17 are conserved across species, their functions remain obscure. In HEK293 cells, Par14 is present in both the nucleus and the cytosol [122,123]. On the other hand, Par17 appears to localize in the mitochondria [124]. Par14 has been reported to directly associate with DNA *in vivo* [125]. Par14 is also a component of pre-rRNA-protein complexes [126,127] and may play a role in RNA processing in the nucleus.

## 5. Conclusions and Implications for Development of Therapeutics

PPIases from all three subfamilies play biological roles in a number of cellular processes, yet they are non-essential genes. This makes them attractive targets in the treatment of a number of diseases. PPIase inhibitors have been used in the clinic as immunosuppressive agents and hold promise as molecular targets for therapy in a number of diseases [69,128,129,130]. This includes viral infections [131], cancer [64,132,133,134], Alzheimer’s disease [135,136], asthma [105,137], cardiovascular disease [138]. Some well known examples of drugs already used clinically include cyclosporin A, FK506, rapamycin, and tacrolimus [139]. Several FKBP inhibitors such as everolimus, zotarolimus, and temsirolius are in Phase III trials as targets for cell proliferation in cancer treatment and for immunosuppression [140]. Cyclophilins such as cyclophilin A plays an import role in HIV-1 replication and cyclophilins have been used in clinical trials as targets for antiviral therapy in the treatment of HIV-1 and chronic Hepatitis C infection [131]. Pin1 is upregulated in several cancers and has also been targeted for anti-cancer therapy by several laboratories [70,141,142,143]. Since Pin1 controls the mRNA stability of several cytokines and chemokines, it is also an attractive target for pulmonary diseases and diseases relating to the immune system, such as inflammation. However, most PPIase inhibitors developed to date are not specific for a particular pathway, and also have undesired immunosuppressive effects. Recent advances in this area appear promising for the development of inhibitors that have anti-viral and anti-inflammatory potential, but lack the anti-immunosuppressive effects [144,145,146]. A greater understanding of the biological roles of PPIases in biological pathways should allow the development of specific inhibitors that target specific biological pathways.
